# Breast cancer PAINT: a first-in-human, dose-escalation study to determine the safety of Plasma Adjuvant INtra-operative Treatment in breast cancer patients

**DOI:** 10.1186/s12885-025-14153-5

**Published:** 2025-04-22

**Authors:** Audrey Glory, Erica Patocskai, Philip Wong

**Affiliations:** 1 Centre de Recherche du Centre Hospitalier de l’Université de Montréal, Montréal, QC Canada; 2 Département de chirurgie, Centre Hospitalier de l’Université de Montréal, Montréal, QC Canada; 3 Radiation Medicine Program, Princess Margaret Cancer Centre, University Health Network, Toronto, ON Canada; 4 Department of Radiation Oncology, University of Toronto, Toronto, ON Canada

**Keywords:** Non-thermal plasma, Breast cancer, Intraoperative treatment, Local recurrence risk

## Abstract

**Background:**

Non-thermal plasma (NTP) refers to an ionized gas composed of ions, electrons and other reactive agents. The anticancer properties of NTP have been proven in vitro and in vivo. The 10-year local recurrence risk (LRR) in breast cancer patients after breast conservation therapy (i.e., lumpectomy, typically followed by radiation therapy) is still as high as 15–20%. NTP could be used to further treat the tumor bed to reduce the LRR.

**Methods:**

Our primary objective is to determine the safe and tolerable dose of NTP treatment following breast cancer lumpectomy. Our secondary objectives are to assess the safety and tolerability of NTP and to assess the cosmetic effects of NTP treatment in patients with breast cancer. Our exploratory objective is to assess the impact of NTP treatment on cancerous and normal tissues. Patients are followed for up to 3 months after NTP treatment. The patients are divided into 3 groups: group A (*n* = 3): NTP treatment of part of the tumor bed ex vivo. Group B (*n* = 3): NTP treatment of part of the tumor bed in situ (all treated tissues are removed for analysis). Group C (*n* = 6–24): dose escalation per “3 + 3 Design” up to a maximum dose level of 3. NTP treatment of part of the tumor bed in situ (the treated parts of the tumor bed will not be excised, except for a small portion for analysis).

**Discussion:**

The safety and tolerability of treatment will be evaluated by means of dose-limiting toxicity, adverse event (AE) and serious adverse event reports; physical examinations; and laboratory safety evaluations. AEs will be coded according to CTCAE v5.0. The results will be tabulated to examine their frequency, grade, and relationship to the study treatment. The results of laboratory assessments will be evaluated similarly. The number of patients with cosmetic alterations linked to NTP treatment and the type of alteration will be assessed through quality of life questionnaires (questions about breast appearance and texture) and through photo collection. This is the first clinical trial to study the safety and tolerability of NTP in an all-breast cancer patient cohort.

**Trial registration:**

Name of the registry: ClinicalTrials.gov. Trial registration number: NCT06222788. Date of registration: 01/15/2024. URL of trial registry record: https://clinicaltrials.gov/study/NCT06222788.

## Background

Surgery is one of the pillars of breast cancer treatment and can even be the only treatment needed in the management of early-stage breast cancer. In the last two decades breast-conserving surgeries (BCS) or lumpectomies have been used more often than radical mastectomies. BCS aim to remove as little tissue as possible to eliminate the tumor while preserving most of the breast. Indications for BCS include ductal carcinoma in situ and T1-2 tumors (if there are no contraindications to adjuvant radiotherapy (RT)) and small tumors that can be resected with clear margins. However, the ratio of tumor to breast size must be considered, and women with larger breasts may still be eligible for BCS despite a larger tumor. Absolute contraindications to lumpectomy include prior RT and inability to obtain clear margins [1]. The use of adjuvant radiotherapy has been proven to decrease the local recurrence risk (LRR) and increase overall survival [2–4]. During lumpectomies, RT is used to complement more conservative cancer surgeries to maintain high local control without resorting to complete mastectomy [5]. Adjuvant RT can be given intraoperatively or postoperatively. Intraoperative radiation consists of inserting a radiation device inside the surgical cavity, and radiation is applied over 20–50 min [6]. External beam radiation therapy is often used to supplement intraoperative radiation therapy. Adjuvant external beam RT is the most common standard form of radiation treatment used to reduce LRR. This treatment consists of treating the entire or a large volume of the breast with radiation therapy over 5–25 days of daily treatment. In this case, a large volume of normal tissue surrounding the surgical cavity is irradiated to annihilate microscopic tumor cells that may permeate centimeters from the visible tumor [7]. As the cancer cell load is highest in the peritumoral area [8, 9], it dictates the dose of RT needed to eradicate the last cancer cells to obtain local control. However, RT can be associated with cosmetic and local adverse events such as breast shrinkage, induration, telangiectasia and breast edema [10–13], as well as severe side effects, such as symptomatic rib fracture, symptomatic lung fibrosis, ischemic heart disease and an increased risk of secondary cancers [10, 11, 13–15]. Moreover, LRR is still as high as 12% for triple-negative breast cancer (TNBC) patients (within 7 years) [16]. Another current strategy for reducing LRR is to use cavity shave margins (i.e., to remove a larger margin of normal tissue from the tumor bed) [17–19]. Cavity shaving, while efficient at reducing the rate of positive margins and the need for re-excision, goes against the current efforts to remove as little normal tissue as possible and is not always desirable when the cavities are close to blood vessels or the chest wall [17].

Plasma is the fourth state of matter; it is a gaseous mixture of electrons, ions, radicals, radiation, etc. Non-thermal plasma (NTP) can be generated with the help of an electric field applied to a gas. The temperature of an NTP usually ranges between 27 °C and 77 °C [20]. When in contact with ambient air, NTP can produce a mixture of reactive oxygen and nitrogen species (RONS) [21, 22], which are cytotoxic to various cancer models [23, 24]. For the treatment of cancer, NTP can be applied directly to cells to increase intracellular RONS levels, inducing damage to DNA, lipids, and proteins, and preventing cell proliferation [25, 26]. The NTP source used for this trial is the convertible plasma jet (CPJ). It has been successfully tested in vitro [27, 28] and in vivo (*to be published*) on breast cancer and sarcoma cell lines.

We hypothesize that NTP could be used intraoperatively, immediately after lumpectomy, to treat the tumor bed. Direct treatment using an NTP jet (where the NTP is in direct contact with the cells) could lead to the killing of remaining cancer cells within and around the surgical bed. Reducing the tumor burden would allow for a reduction in the LRR. In previous clinical [29, 30] and in vivo [31, 32] studies, NTP has not been shown to be associated with any severe adverse events. NTP could therefore be used instead of or in combination with the current methods to reduce LRR, for example by decreasing the dose of RT needed to clear the tumor bed of microscopic tumor cells left after surgery. The intraoperative use of NTP would not be an extra step for the patient, as it would be administered by the surgeon during the surgery. Before that technology can be assessed for its clinical efficacy, the safety and toxicity of NTP produced by CPJ must be investigated in humans. Therefore, this trial aims to determine safe and tolerable CPJ treatment conditions following breast cancer lumpectomy.

## Methods and study design

This first-in-human study was approved by the University of Montreal Research Hospital (CHUM) Research Ethics Board (#23.167) in compliance with the Declaration of Helsinki.

### Objectives

- Primary objective: To determine the safe and tolerable dose of NTP in patients with breast cancer.
  - Primary endpoint: Using a 3 + 3 dose escalation design up to a maximum dose of 3, a safe and tolerable dose of NTP in patients with breast cancer will be determined. The adverse effects of NTP produced by the CPJ will be assessed based on DLTs, AEs, physical exams, quality of life (QOL) questionnaires, and clinically significant changes in laboratory evaluations. The AEs will use the descriptions and grading scales found in the revised NCI Common Terminology Criteria for Adverse Events (CTCAE). This study will utilize CTCAE Version 5.0 for adverse event reporting. All patients that receive the treatment and complete the 1-week follow-up visit will be evaluated for acute toxicity. All patients who receive the treatment and complete the 3-month follow-up visit will be evaluated for long-term events.
- Secondary objectives: To assess the safety and tolerability of NTP in patients with breast cancer and to assess the cosmetic effects of NTP treatment in patients with breast cancer.
  - Secondary endpoints: Number of patients with adverse events linked to NTP treatment and type of adverse event and number of patients with cosmetic alterations linked to NTP treatment and type of alteration. The adverse events of special interest (AESIs) will be assessed through QOL questionnaires (questions on breast appearance and texture) and through photo collection. The following AESIs will be recorded throughout the study:
    - Fatigue; grade 3,
    - Breast pain; grade 3,
    - Skin ulceration; grade 3,
    - Chest wall necrosis; grade 3,
    - Breast atrophy; grade 3,
    - Change in breast texture,
    - Cosmetic issues,
    - Discoloration.
- Exploratory objective: To assess the impact of NTP treatment on cancerous and normal tissues.
  - Exploratory endpoint: Evidence of cancer cell death and absence of normal tissue degradation in samples treated with NTP ex vivo or in situ. The exploratory objective (to assess the impact of NTP treatment on cancerous and normal tissues) will be assessed through the analysis of treated and untreated tissues.

### Subject selection

This trial will be conducted in compliance with the protocol, the ethical principles that have their origin in the Declaration of Helsinki, good clinical practice (GCP), ISO 14155:2020 and Health Canada Medical Devices regulations. Any questions about eligibility criteria must be addressed prior to patient registration.

#### Inclusion criteria

1. Age ≥ 18 years at the time of signing the study consent form.
2. ECOG ≤ 2.
3. Patient with cT1-4 breast cancer for groups A and B; patient with cT1/cT2 breast cancer (based on physical examination, not radiological measurements) for group C. Histological diagnosis must be invasive ductal carcinoma of the breast. 2. Patients are staged per standard of care practice. Patients with the eligible T stage cancers of any N or M stage are eligible.
4. The patient is scheduled to undergo a lumpectomy.

#### Exclusion criteria

1. Prior treatment for the tumor of interest (including chemotherapy, immunotherapy, or radiotherapy).
2. Patient planning or undergoing intraoperative radiotherapy.
3. Diabetes (types I and II).
4. Hypercortisolism.
5. Collagen vascular disease.
6. Patient requiring systemic corticosteroids at physiologic doses exceeding 10 mg/day of prednisone or its equivalent.
7. Patient receiving daily chemotherapy for rheumatological conditions.
8. Pregnancy (a urine pregnancy test must be obtained for nonsterile women of childbearing potential prior to surgery).

### Study investigational medical device – convertible plasma jet

The NTP source used for this trial is the Convertible Plasma Jet (CPJ) from NexPlasmaGen, Inc. A complete description of this NTP applicator can be found in patents No. US 2007/0029500 A1 and US 2022/0353982 A1, as well as in Boisvert et al. [27]. The CPJ is considered a Class III medical device per Health Canada regulations.

Briefly, the electrode configuration of the CPJ device is a coaxial configuration in which a dielectric material separates the ground electrode and the annular open gap for the flowing gas. A quartz tube acts as the dielectric barrier and is placed directly inside the grounded tube, thus leaving an annular gap for gas injection between the dielectric and the central powered tube electrode. The whole assembly forms a dielectric barrier discharge configuration 3 cm in length. As the high-voltage electrode is hollow, the NTP-forming gas can be injected either through it or through the annular gap [27].

With the CPJ, it is possible to sustain three different discharge modes (Ω; 𝛾 and jet modes) when a 13.56 MHz sinusoidal excitation waveform is fed to the high-voltage electrode. For this study, all the treatments will be performed at atmospheric pressure (101.3 kPa) with helium (99.999% purity) injected within the annular gas gap between the dielectric and the hollow high-voltage electrode and with oxygen (medical grade) injected within the high-voltage electrode. The discharge mode used will be the 𝛾 mode.

### Rationale for the starting dose and dosing schedule

The dose of NTP produced by the CPJ is described by the duration (min) of the treatment. Based on the in vivo studies performed with the CPJ V4.4, the maximum dose used will be 5 min of treatment at 13 W (0 Hz, 100% DC), 5000 sccm helium and 25 sccm O_2_ per 7 mm^2^. This dose has proven to be efficacious and safe in mice (*manuscript in preparation*). For V5 (clinical version) of the CPJ, by comparing the physical, electrical and thermal properties of V4.4 and V5, the equivalent conditions were found to be 220 V, 100 Hz, 80% DC, 5000 sccm helium and 20 sccm O_2_ in the capillary, with stationary treatment for 5 min per 7 mm^2^. This dose will be defined as the highest dose assessed in this trial, dose 3 (Table 1). The dose will not be increased above this mark in search of the maximum tolerated dose (MTD), as it would not necessarily be associated with improved treatment efficacy (probably due to the increase in temperature that accompanies the increase in applied power). Instead, the maximum dose used in this study will be the dose that has been found to be efficacious (i.e., dose 3) in vivo or lower in the presence of dose-limiting toxicities (DLTs). A DLT is defined as any of the following attributable to NTP administration (definitely, probably, or possibly) occurring after NTP administration until the 1-week post-op follow-up visit:

- Fever; grade 4,
- Breast infection; grade 4,
- Skin ulceration; grade 4,
- Chest wall necrosis, grade 4.

**Table 1:** Doses used in the breast cancer PAINT first-in-human investigation

Dose Level #	Dose	Group
**Dose Level − 1**	**2 min per 7 mm** ^**2**^	**Group C – if 1 DLT out of 3 or 2 DLTs out of 6 (30 min max)**
Dose Level 1 (starting dose)	3 min per 7 mm^2^	Group C, Cohort 1 (30 min max)
Dose Level 2	4 min per 7 mm^2^	Group C, Cohort 2 (30 min max)
Dose Level 3	5 min per 7 mm^2^	Group C, Cohort 3 (30 min max) Group A (15 min max) Group B (15 min max)

Three dose levels will be assessed for DLTs. If DLT toxicity occurs in the initial cohort for group C, then a provision has been added for a fourth lower dose to be explored with an expanded patient cohort (in bold text)

### Treatment plan

The minimum number of patients required to complete this first-in-human study will be 9, and the maximum sample size for the study will be 30. At the end of each group/cohort, a safety cohort review will be performed to analyze the safety data and approve the transition to the next group/cohort if applicable.

The study will be conducted at the CHUM site in Montreal, Quebec.

#### Group A (*n* = 3)

**Fig. 1:**
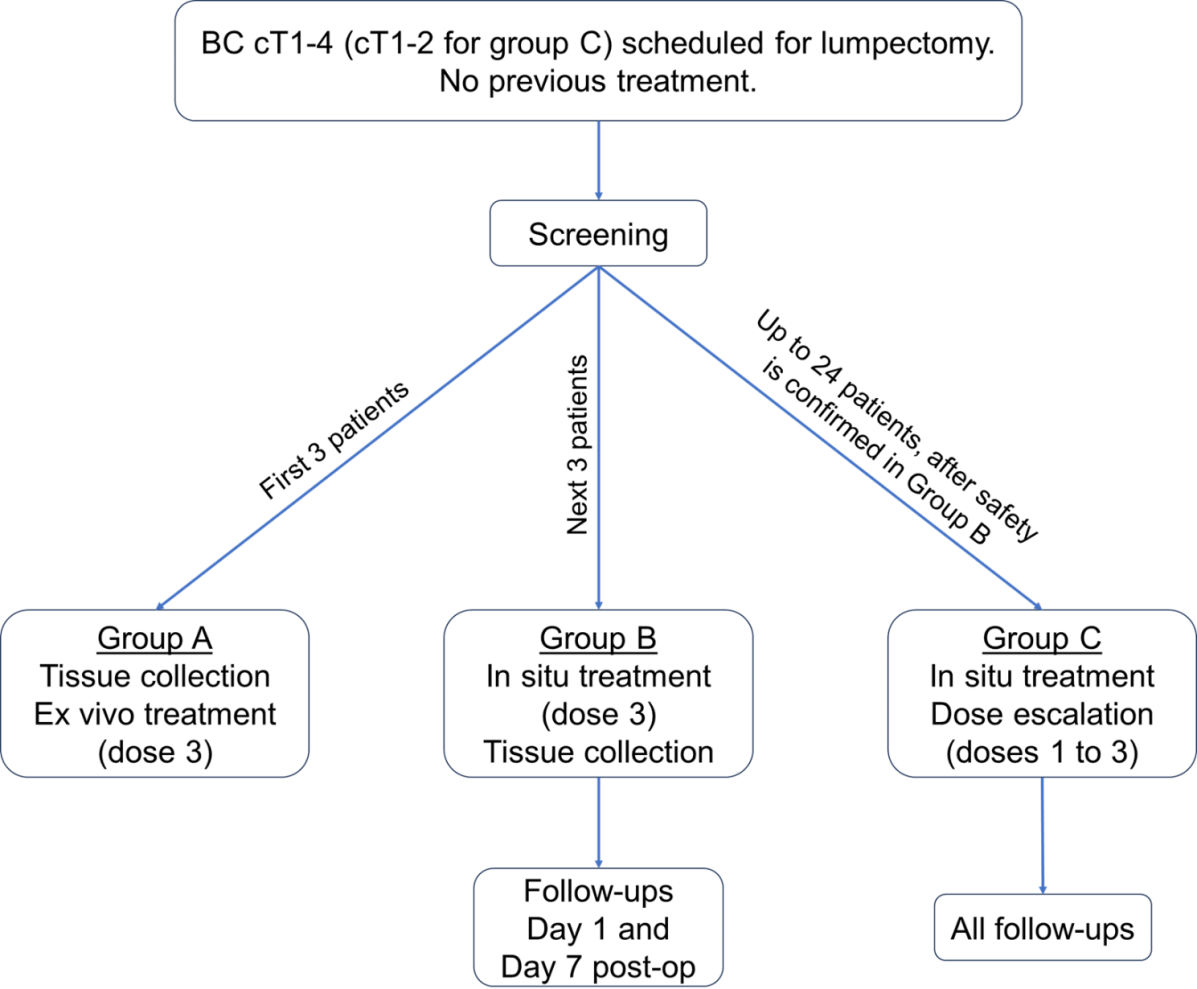
Study schema. BC = breast cancer. Simplified representation of the proceedings of the breast cancer PAINT study

- All three subjects can be enrolled concurrently. These patients will be treated concomitantly as the treatment will take place ex vivo. Once three subjects are enrolled in group A, it will be closed to enrollment. The enrollment of group B patients can start immediately (Fig. 1).

- Treatment of part of the tumor and tumor bed ex vivo will be performed at dose 3 (5 min per 7 mm^2^) for a maximum of 15 min (i.e., treatment of 21 mm^2^). During surgery, the tumor and the tumor bed will be removed. Part of the tumor and tumor bed will be reserved for NTP treatment, while the rest will be sent to pathology as part of the standard of care (SOC) procedures. All tissues exposed to NTP will be analyzed.

#### Group B (*n* = 3)

- Three patients in group B will then be enrolled concurrently and treated with NTP, also concomitantly, as none of the NTP-treated tissues will be left inside the patients. Group B will then be closed to enrollment until safety assessment of all three subjects is performed at post-op follow-up #2. Safety will be confirmed if none of the three patients experience DLTs (Fig. 1).
- NTP treatment of part of the tumor bed in situ at dose level 3 (5 min per 7 mm^2^) for a maximum of 15 min (i.e., treatment of 21 mm^2^). During surgery, part of the tumor bed will be treated with NTP inside the patient. The treated tumor bed will be removed from the patient immediately after NTP treatment and analyzed as part of this clinical protocol. Part of the tumor removed during surgery will be sent to the PW laboratory for treatment with NTP at dose 3 and analysis as part of this clinical protocol. The rest of the tumor and tumor bed will be sent to pathology as part of the SOC procedures. No treated tissue will stay inside the patient.

#### Group C (*n* = 6–24)

**Fig. 2:**
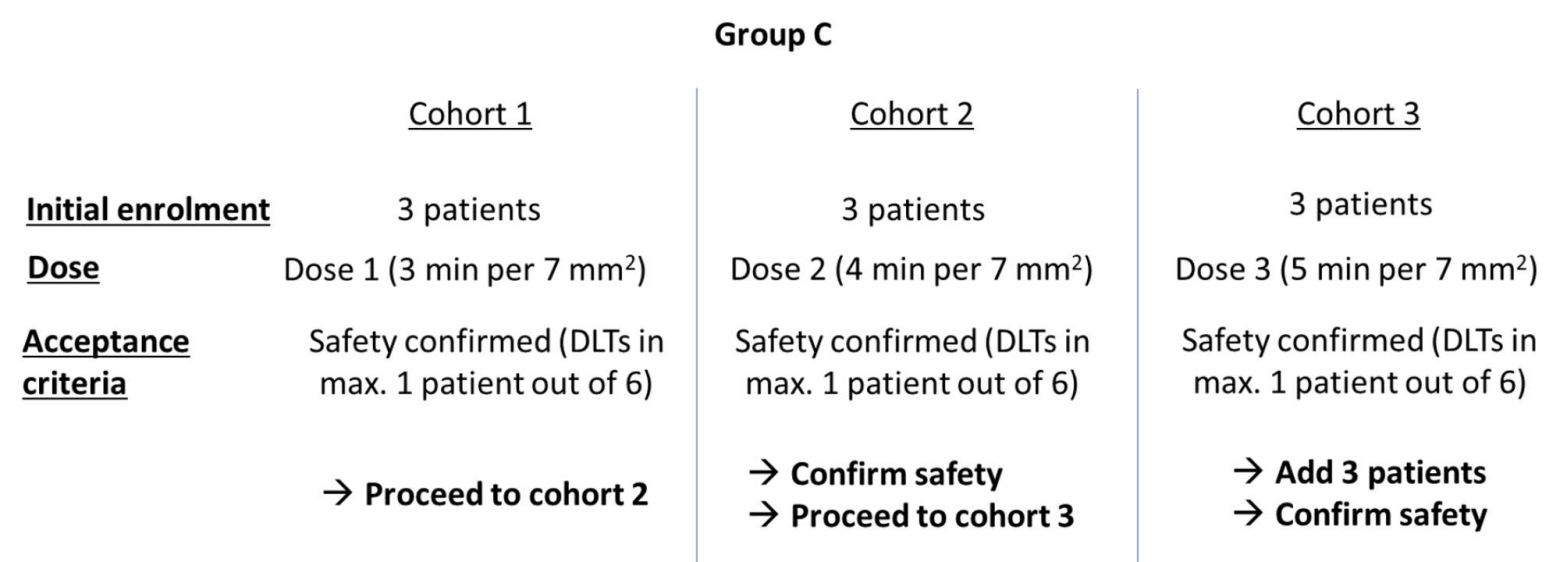
Schematic of the clinical plan for group C. Details of the distribution of patients in group C in cohorts 1, 2 and 3, with associated dose and acceptance criteria

- After safety is confirmed for group B, group C patients will be enrolled based on the 3 + 3 dose escalation rule up to a maximum dose level of 3 (Table 1). Even if the safety of dose 3 is confirmed in group B, the first patient cohort treated in group C will receive a lower starting dose of 3 min per 7 mm^2^ (dose 1) (Table 1). In cohort 1, at dose level 1, the first patient will be enrolled and treated with NTP. After follow-up #2 one-week post-op, only if there was no DLT in the first patient could accrual be reopened to enroll the second and third patients in the cohort. Subjects will be enrolled in cohorts of three each. If no DLT is encountered in any of the 3 subjects after 1-week post-op, dose escalation according to Fig. 2 will be allowed. If one of the 3 subjects experiences a DLT, three additional subjects will be enrolled at the same dose level, and if none of these 3 additional subjects experience a DLT, dose escalation will be allowed. A safety cohort review will be conducted after the patients in each cohort complete the 1-week post-op follow-up visit. The MTD will be defined as the next lower dose level below the one in which > 1/3 of the subjects or ≥ 2/6 of the subjects experience DLT. Up to 6 additional subjects may be enrolled at the MTD to obtain additional data on the safety and efficacy of the product. If escalation to dose level 3 is reached and < 1 out of 6 subjects experience DLT, dose escalation will stop, and 6 additional subjects will be enrolled at dose level 3 to obtain additional data on the safety and efficacy of the product.

- One NTP treatment will be conducted in the tumor cavity after the tumor is removed, at three dose levels (1, 2 and 3). The NTP starting dose is dose level 1, for 30 min maximum (i.e., treatment of 42 mm^2^). The treated parts of the tumor bed will not be excised, except for a small portion for analysis. A small portion of the tumor removed during surgery will be treated ex vivo in the PW laboratory.

Given that the safety of the treatment is related to the duration of exposure of normal tissues, we established a maximum NTP treatment duration of 30 min per patient. At each dose, NTP treatment of the prespecified duration will be applied to the region with the highest risk of harboring residual cancer cells based on the surgeon’s judgment. This region will be identified on the tissue before treatment with 4 surgical threads. The size of the zone treated with plasma will be 21 mm^2^ for groups A and B. For group C, the size of the treated zone will stay the same across cohorts (i.e. 42 mm^2^); only the duration of treatment will change. For cohort 3, at dose 3 (5 min per 7 mm^2^), the size of the surface of the tissue that can be treated in 30 min is 42 mm^2^, representing a 3 mm × 14 mm rectangle (3 mm being the diameter of the plasma). For cohort 2, at dose 2, this 42 mm^2^ rectangle will be treated for 24 min, and for cohort 1, at dose 1, it will be treated for 18 min. NTP will be applied with a slow sweeping motion, at an approximate speed of 3 s per 21 mm^2^. The orientation and direction of the NTP will be up to the surgeons, to allow for practical implementation of the tool during the operation.

### Study calendar

The details of the visits associated with this study and their content can be found in Table 2.

**Table 2:** Study calendar

Required Investigations	Screening	Day of Surgery and NTP treatment	Post-op Follow-Up #1^1^	Post-op Follow-Up #2	Post-op Follow-Up #3	End of study Follow-up	
Window	Within 28 days of treatment	-	Day 2–3 post-op	Day 7 post-op; +/- 3 days	Day 30; +/- 7 days	Day 90; +/- 30 days	
Demographics	X						
History and Physical Exam	X	X	X	X	X	X	
Quality of life questionnaire	X^2^		X	X	X	X	
Hematology, Biochemistry	X				X	X	
NTP administration and Tissue collection		X					
Ultrasound	X				X		
Image collection	X^3^			X	X	X	
ECG	X						
Concomitant medication review	X	*Continuously*					
Adverse events	*Continuously*						

^1^ Post-op follow-up #1 can be conducted by phone. If so, only ECOG performance status and quality of life questionnaires will be mandatory

^2^ Not applicable for group A

^3^ Image collection for group C patients only

**Fig. 3:**
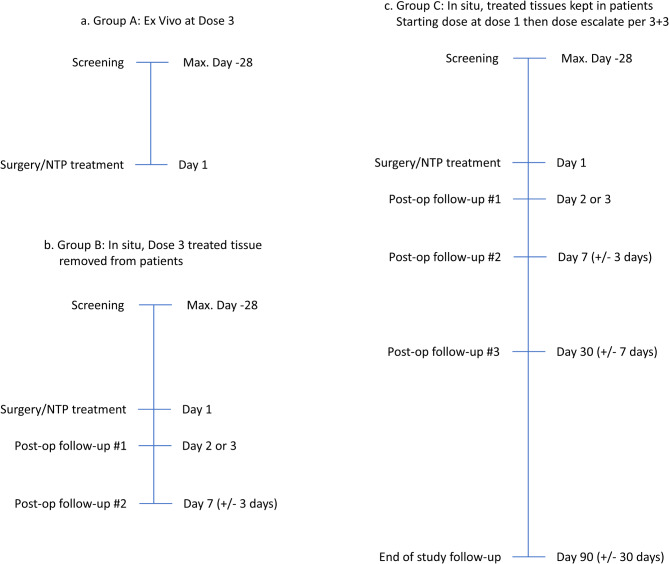
Study timelines for each group. These three schemes detail the patient’s journey in (**a**) group A; (**b**) group B and (**c**) group C

The follow-up period covers 3 months and is divided into 4 follow-up visits or calls. The first follow-up visit is at 2–3 days post-op, with a physical exam and a QOL questionnaire (custom-made for this study). The second follow-up is at 7 days post-op, with a physical exam and a QOL questionnaire for groups B and C, and the image collection for group C only. The third follow-up is at 30 days post-op, with a physical exam, a QOL questionnaire, blood tests (hematology and biochemistry), ultrasound and image collection. The last follow-up is at 90 days post-op, with a physical exam, a QOL questionnaire, blood tests (hematology and biochemistry) and image collection. Group A (ex vivo only) does not have to attend any follow-up visits (Fig. 3a). The participation of group B in this study stops after follow-up #2 (Fig. 3b). Patients in group C must attend all the visits (Fig. 3c).

### Image collection (for group C patients only)

One picture of the area of the surgery, showing about 20 cm in diameter around the scar (or the intended location of the scar for the pre-surgery picture). Cutaneous changes (other than the scar itself) will be compared between the pre-surgery picture and the post-surgery/NTP pictures. The changes will be scored on a four-point graded scale from 0 to 3 (0 = no change; 1 = mild changes, intervention not indicated; 2 = moderate changes, minimal, local or noninvasive intervention indicated; 3 = severe or medically significant, extensive or systemic intervention indicated). All the images will be scored by three independent observers. If necessary, historical results will be used to compare with results obtained from this trial.

### Tumor collection and correlative studies

For all groups, untreated tumor and normal tissue will be sent from the operating room to pathology according to standard procedure. In pathology, a portion of the untreated tumor and normal tissue will be given to a member of PW’s team for correlative studies.

For groups B and C, the entire treated tumor bed (group B) or a small portion of the treated tumor bed (group C) will be divided between pathology and PW’s laboratory.

The tissues that stay in pathology will be fixed, embedded in paraffin, cut into slices and mounted on glass slides following the CHUM’s protocols. The hematoxylin and eosin (H&E) coloration technique will be used to reveal the microscopic anatomy of the tissue. The goal is to obtain insight into the effect of NTP on cancer cells (tumor tissue) and normal cells (tumor bed tissue) immediately after NTP treatment. H&E staining allows visualization of potential damage to tissues.

For the untreated samples going into PW’s laboratory, they will be separated into six equal parts:

- Three parts for treatment ex vivo and immediate fixation: one will stay untreated; one will be treated with gas only and one will be treated with the same NTP parameters used in this trial. After treatment, they will be immediately fixed and paraffin-embedded to document the immediate effects of NTP ex vivo using H&E staining.
- Three parts for treatment ex vivo and delayed fixation: one will stay untreated; one will be treated with gas only and one will be treated with the same NTP parameters used in this trial. After treatment, they will be incubated in Dulbecco’s Modified Eagle Medium (DMEM) at 37 °C in a humidified atmosphere of 5% CO_2_ for 24 h to document the long-term effect of NTP. After 24 h, the samples will be fixed and paraffin-embedded. Histological tests and immunofluorescence staining will be performed, including TUNEL for apoptosis, y-H2AX for DNA damage, KI-67 for proliferation and acrolein for oxidative stress. The in situ-treated samples going into PW’s laboratory will follow the same protocol and be analyzed concurrently.

All the tissue slides produced during this study will be blindly analyzed by two pathologists who will also be blind to each other’s assessments.

### Statistical analysis

The following study populations are defined and will be analyzed as specified below. The population evaluable for safety will be the safety population.

The Intent to Treat population: 24 patients.

Safety population: up to 30 patients.

Per protocol population: any patient who received plasma treatment within group C.

The study population will consist of all patients who are registered and who received one dose of NTP. All analyses will be conducted using the study population. Any patient who is registered for this trial but never receives study treatment will be described, including the reason(s) for nonparticipation.

Subject characteristics will be described in a table comprising age, tumor type and grade, and Eastern Cooperative Oncology Group (ECOG) status. Subjects will be summarized using their group/cohort.

Summary statistics will be used to describe baseline characteristics and other outcomes of interest. Categorical endpoints will be summarized using proportions and frequencies. Continuous endpoints will be summarized using the mean, median, range or standard deviation. Subgroup summarization based on dose level or other criteria may also be conducted.

### Statistical analysis methods

Evaluable patients (all patients who started treatment) will be included in the final analysis. The usual components of this analysis are as follows:

- Tabulation of all patients entered, and any patients excluded from the analysis with reasons for exclusion;
- Patient accrual rate;
- Distribution of important baseline prognostic variables;
- Frequency and severity of adverse events;
- Observed results with respect to the endpoints described above.

Any deviation from this statistics section of the protocol, along with the accounting for missing, unused and spurious data, will be described in the final report.

## Discussion

This first-in-human trial aims to determine safe and tolerable CPJ treatment conditions following breast cancer lumpectomy. If successful, the CPJ, an NTP-producing device, could be used intraoperatively immediately after lumpectomy to treat the tumor bed in a phase II clinical trial to investigate the efficacy of NTP produced by the CPJ.

Two similar NTP devices had previously been tested in phase I trials. The kINPen Med was used in two studies on twelve and six patients respectively, suffering from treatment-resistant locally advanced oropharyngeal cancer [29, 33]. These studies showed that the kINPen Med improved QOL, reduced contamination and decreased patients’ need for pain medication. A decrease in tumor size was observed in four out of twelve patients and two out of six patients, respectively. There were no deaths related to NTP. The side effects included fatigue, dry mouth-like symptoms, dysgeusia, pain, bleeding and local edema [29, 33]. NTP treatment did not prolong overall survival [29]. The Canady Helios cold plasma (CHCP) [34] was tested in a stage IV metastatic colon cancer patient [35] and in a basket phase I trial [36]. The phase I trial (ClinicalTrials.gov identifier NCT04267575) evaluated the application of CHCP-produced NTP to the surgical margin and macroscopic tumor sites of patients with stage IV metastatic or recurrent solid tumors [36]. Twenty patients were recruited with 16 different types of cancer who had received prior cancer therapies (surgery, chemotherapy, radiotherapy or hyperthermic intraperitoneal chemotherapy; neoadjuvant or adjuvant). There were no intra- or postoperative complications [36]. Ex vivo-treated normal tissue showed no signs of thermal damage or histological changes. There were no adverse events attributable to CHCP or NTP [36]. The cosmetic aspect of the treatment was not explored. Moreover, a prospective, single-armed phase IIb trial was conducted on 20 patients with cervical intraepithelial neoplasia (CIN) 1 (mild dysplastic lesions) or 2 (moderate dysplasia) using the VIO® 3, APC 3 NTP-producing device (ClinicalTrials.gov identifier NCT03218436). Transmucosal tissue devitalization was achieved ex vivo and in vivo. After a 24-week follow-up period, 19 out of 20 (95%) participants achieved complete remission. No postinterventional complications were reported, other than mild to moderate discomfort during NTP application [37].

Direct treatment using an NTP jet (where the NTP is in direct contact with the cells) could lead to the killing of remaining cancer cells within and around the surgical bed. As reducing the tumor burden would allow for a reduction in the LRR, NTP could be used instead of or in combination with the current standard methods to reduce LRR. As several studies have demonstrated NTP’s ability to induce immunogenic cell death [38, 39], future development could include assessing NTP in combination with immune modulation for cancer treatment [40].

## Data Availability

No datasets were generated or analysed during the current study.

## Abbreviations

AE: Adverse Event
AESI: Adverse Event of Special Interest
BC: Breast cancer
BCS: Breast conserving surgery
°C: Degrees Celsius
CHCP: Canady Helios Cold Plasma
CHUM: Centre Hospitalier de l’Université de Montréal (University of Montreal Research Hospital)
CIN: Cervical Intraepithelial Neoplasia
Cm: Centimeter
CO_2_: Carbon dioxyde
CPJ: Convertible Plasma Jet
CR-CHUM: Centre de Recherche du Centre Hospitalier de l’Université de Montréal (research center of the University of Montreal Research Hospital)
CTCAE: Common Terminology Criteria for Adverse Events
DC: Duty cycle
DLT: Dose-limiting toxicity
DMEM: Dulbecco’s Modified Eagle Medium
DNA: Deoxyribonucleic Acid
ECG: Electrocardiogram
ECOG: Eastern Cooperative Oncology Group
GCP: Good clinical practice
H&E: Hematoxylin and Eosin
He: Helium
Hz: Hertz
kPa: Kilo Pascal
LRR: Local Recurrence Risk
Max: Maximum
Mg: Milligram
MHz: Mega Hertz
min: Minute
mm: Millimeter
mm^2^: Square millimeter
MTD: Maximum Tolerated Dose
NTP: Non-Thermal Plasma
O_2_: Dioxygen
PAINT: Plasma Adjuvant Intraoperative Treatment
QOL: Quality of Life
RONS: Reactive oxygen and nitrogen species
RT: Radiotherapy
sccm: Standard cubic centimeters per minute
SOC: Standard of Care
TNBC: Triple-negative breast cancer
TUNEL: Terminal deoxynucleotidyl transferase dUTP nick-end labeling
V: Volt
V4.4: Version 4.4
V5: Version 5
W: Watt
